# Peer-Led Emotional CPR Program in Black, Indigenous, and People of Color Communities: Convergent Mixed Methods Study

**DOI:** 10.2196/80029

**Published:** 2026-05-28

**Authors:** Mbita Mbao, Karen L Fortuna

**Affiliations:** 1 School of Social Work, Salem State University, 352 Lafayette Street, Salem, MA, 01970, United States, 1 9785900954; 2 Patient Innovation Lab, Department of Community and Family Medicine, Geisel School of Medicine at Dartmouth, Dartmouth College, Lebanon, NH, United States

**Keywords:** Emotional Connection, Empowering, Revitalizing, Emotional CPR, peer support, Black, Indigenous, and people of color, mental health education, trauma-informed care, empowerment, loneliness, mixed methods research, cultural safety

## Abstract

**Background:**

Black, Indigenous, and people of color (BIPOC) experience disproportionately negative mental health outcomes, including rising suicide rates, and persistent barriers to culturally responsive care. Systemic racism, discrimination, and historical trauma contribute to mistrust of traditional mental health systems. Peer-led approaches that center lived experience and shared cultural identity may offer culturally responsive alternatives that foster emotional connection and trust.

**Objective:**

This study examined pretraining to posttraining changes in emotional well-being, empowerment, and interpersonal outcomes among BIPOC participants who completed Emotional CPR (Emotional Connection, Empowering, Revitalizing), a trauma-informed, peer-led mental health education program facilitated by BIPOC trainers.

**Methods:**

A convergent mixed methods pre-post design was used. Eighty-five BIPOC participants completed validated self-report measures immediately before and after a 12-hour web-based Emotional CPR training delivered over 3 consecutive days. Outcomes included loneliness, empowerment, flourishing, hope, active-empathic listening, mindfulness, social connectedness, and affect. Paired-samples 2-tailed *t* tests examined pre-post differences. Seventeen participants participated in 2 posttraining focus groups conducted through Zoom. Qualitative data were analyzed using thematic analysis informed by phenomenological principles. Quantitative and qualitative findings were integrated during interpretation.

**Results:**

Participants (N=85; mean age 41.9, SD 11.2 years; women: n=65, 76.5%) reported significant reductions in loneliness (*P*=.03; *d*=0.25) and significant increases in positive affect (*P*=.002; *d*=–0.37). Significant declines were observed in empowerment (*P*=.03; *d*=0.26), active-empathic listening (*P*=.02; *d*=0.26), flourishing (*P*=.02; *d*=0.27), and hope (*P*<.001; *d*=0.41). No significant changes were observed in social connectedness or mindfulness. Qualitative themes included empowerment through skill-building, emotional vulnerability, cultural trust and safety, challenges in sustaining confidence after training, and the application of skills in daily life. Participants described increased self-awareness and emotional openness, particularly within a culturally responsive training environment. Qualitative findings suggested that increased emotional awareness may have influenced posttraining self-assessments.

**Conclusions:**

Participation in BIPOC-facilitated Emotional CPR training was associated with short-term reductions in loneliness and increased positive affect, alongside modest declines in self-rated empowerment and hope. Qualitative findings suggest that increased emotional awareness and engagement with systemic stressors may temporarily influence self-perceived competence. Ongoing reinforcement, mentorship, and follow-up sessions may support sustained empowerment and emotional resilience in culturally responsive peer-led interventions.

## Introduction

Black, Indigenous, and people of color (BIPOC), a term used to describe individuals who identify as non-White and are often marginalized within dominant racial hierarchies, continue to face disproportionately negative mental health outcomes [1]. From 2018 to 2023, Black individuals and American Indian or Alaska Native communities experienced the most significant increases in suicide rates [2]. Despite the need, BIPOC individuals continue to face barriers to accessing behavioral health services [3]. Even when care is received, discrimination from providers can contribute to poorer outcomes [3]. These inequities, rooted in systemic racism, historical trauma, and mistrust of the mental health system, underscore the importance of culturally responsive support systems [1,4].

A growing body of research highlights the value of peer-to-peer models in mental health recovery, especially within BIPOC communities [5]. Prior studies demonstrate that peer-led interventions can reduce social isolation, increase engagement in care, and promote empowerment by centering lived experience and shared identity [5-8]. Recent qualitative evidence further demonstrates that peer-led groups promote relational safety and meaning-making through shared lived experience, which participants identify as a central mechanism of change [6]. These approaches are particularly promising in communities that have experienced historical marginalization and mistrust of traditional mental health systems [7,8].

In this context, we sought to examine whether participation in an Emotional CPR (Emotional Connection, Empowering, Revitalizing) intervention was associated with changes in emotional well-being, empowerment, and interpersonal skills. Emotional CPR is a peer-developed, trauma-informed mental health education and training program designed to equip community members with the skills and confidence to support individuals experiencing emotional distress or crisis [9-11]. Grounded in the recovery model, Emotional CPR emphasizes a person-centered approach that prioritizes lived experience, supportive relationships, emotional safety, and trust, and it is intended for application in community-based nonclinical settings [12]. Although Emotional CPR shares similarities with community-based interventions such as Mental Health First Aid, which equips laypersons to recognize and respond to mental health crises, the 2 approaches differ in emphasis. Mental Health First Aid focuses on symptom recognition, risk assessment, and referral pathways [13], whereas Emotional CPR centers on emotional presence, relational connection, and trauma-informed engagement.

Rather than concentrating on identifying disorders or directing individuals toward formal services, Emotional CPR emphasizes “being with” people in distress, fostering mutual emotional connection, and supporting empowerment within community contexts [9]. This relational and recovery-oriented orientation distinguishes Emotional CPR from more clinically structured mental health literacy models and aligns it more closely with peer-support frameworks grounded in lived experience. Within this study, recovery is conceptualized as a person-centered process that emphasizes hope, empowerment, supportive relationships, and the capacity to live a meaningful life beyond symptom reduction [12,14].

To further support this recovery framework, the intervention was intentionally designed to be delivered by BIPOC trainers to support culturally responsive and community-centered engagement. This approach aimed to cultivate cultural safety and mutual understanding and to reduce the burden on participants to explain or justify their experiences. Creating a supportive environment that fosters personal growth, strengthens resilience to stress and adversity, and encourages cultural and spiritual development is central to recovery [14]. Feeling validated, heard, and understood by trusted community members, as well as by health and social service providers, may be beneficial for individuals navigating emotional distress and working toward sustained well-being [5,14].

This study examined the pretraining to posttraining changes among BIPOC participants who completed an Emotional CPR training facilitated by BIPOC trainers. Using a convergent mixed methods design, we assessed changes in participants’ emotional well-being, empowerment, and interpersonal outcomes, alongside their lived experiences of the training. By focusing on culturally responsive, peer-led spaces, this study contributes to efforts to strengthen culturally responsive mental health supports for BIPOC communities.

## Methods

### Study Design

This study used a convergent mixed methods pre-post design, in which quantitative and qualitative data were collected during the same study phase, analyzed separately, and integrated during interpretation. The quantitative component assessed changes in self-reported well-being, empowerment, and social connectedness through validated psychological measures. In contrast, the qualitative component explored participants’ experiences and perceptions through focus group discussions. The integration of both data types allowed for a deeper exploration of the training’s effects and areas for future improvement.

### Description of Emotional CPR Training

Emotional CPR is designed to be facilitated by individuals with lived experience of mental health challenges, including anxiety, depression, bipolar disorder, schizophrenia, and trauma. Facilitators may include peer support specialists, community members, and professionals such as educators, administrators, and first responders. The emphasis on lived experience and relational understanding reflects the program’s grounding in peer support and recovery-oriented principles.

The Emotional CPR training is a 12-hour web-based course delivered over 3 consecutive days through live, synchronous videoconference sessions (Zoom). The curriculum includes the following modules: (1) establishing emotional connection, (2) recognizing and utilizing nonverbal communication, (3) fostering cultural empathy across different worldviews, (4) applying trauma-informed practices, (5) responding to emotional distress and suicidal ideation, (6) promoting empowerment, and (7) fostering revitalization.

Sessions combine didactic instruction with facilitated group dialogue, structured real-play exercises conducted in breakout rooms, and guided reflection activities. Modules are sequenced to move from foundational concepts of emotional connection and trauma-informed engagement toward applied practice in cultural empathy, responding to distress, empowerment, and revitalization. Experiential learning is integrated throughout the training to reinforce skill development and encourage reflective practice.

### Training Facilitators

Trainers facilitating Emotional CPR complete a structured 60-hour certification program that includes experiential training in trauma-informed engagement, cultural humility, crisis response, and supervised practice sessions, and they must demonstrate competency through evaluated real-play and a final assessment prior to certification [10,11]. In this study, a total of 7 certified trainers facilitated the sessions. Each training cohort included approximately 6 to 16 participants, with smaller breakout groups used for real-play and discussion exercises to support interactive learning and engagement.

Facilitators included individuals with lived experience of mental health challenges, as well as professionals such as peer support specialists, educators, and community-based practitioners. Facilitators identified as BIPOC, including Black/African American, Native American, and multiracial individuals. Many facilitators had personal and professional lived experience within the mental health system, including roles in peer support and community-based services, which informed their facilitation approach. Cultural relevance was not assumed solely on the basis of shared racial identity. Instead, it was operationalized through culturally responsive facilitation practices, including cultural humility, validation of diverse lived experiences, and the intentional creation of a space where participants could engage without needing to explain or justify culturally specific experiences. Facilitators drew on their lived experience and community-based knowledge to support relational engagement and trust-building.

Participants were recruited online through community-based networks and were not restricted by geographic location. As a result, participants likely represented diverse regional and cultural contexts within the United States; however, geographic data were not systematically collected. This limits the ability to assess the influence of shared place-based experiences on culturally responsive engagement within the training environment.

### Ethical Considerations

The study was approved by the WCG Institutional Review Board (application number 1284189). All participants provided electronic informed consent prior to participation. Participation was voluntary, and participants could withdraw at any time without penalty. Data were deidentified prior to analysis and stored on secure, password-protected systems. Participants were not compensated for their participation.

### Participants and Recruitment

Participants were recruited through community-based outreach efforts, including email listservs, social media announcements, and partnerships with community organizations serving BIPOC populations. Recruitment materials described the Emotional CPR training as a peer-led mental health education opportunity facilitated by BIPOC trainers. Interested individuals self-enrolled in the training through an online registration platform. Inclusion criteria required participants to (1) identify as Black, Indigenous, or a Person of Color and (2) be 18 years of age or older. Participants were not required to have lived experience with mental health challenges to enroll in the training, as the program is designed for a broad audience, including community members, individuals with lived experience, and professionals seeking to strengthen their capacity to support others in emotional distress. Lived experience with mental health challenges was not systematically assessed as part of the study. Participants were recruited online and were not restricted by geographic location; however, geographic data were not systematically collected, which limits the interpretation of shared cultural context related to place.

Of those enrolled in the training, 85 participants completed both presurvey and postsurveys. For the qualitative component, purposive sampling was used to invite survey completers to participate in focus groups. Invitations were distributed through email following training completion, and participants were selected based on availability and willingness to ensure diverse representation across gender and racial identities. A total of 17 participants participated in 2 focus groups (n=7, 41%; n=10, 59%).

### Quantitative Data Collection

Participants completed a battery of validated psychological measures at 2 time points: immediately prior to the first training session (pretraining) and immediately following the completion of the third training day (posttraining).

### Validated Psychological Measures

#### Herth Hope Scale

The Herth Hope Scale was used to measure hope across 3 dimensions: inner temporality and future, inner positive expectancy, and interconnectedness with others. This 12-item scale is reliable and valid for both older and chronically ill populations, with strong internal consistency (*α*=.84-.97) [15]. Sample items include “I have a positive outlook toward life,” “I have short- and/or long-range goals,” and “I feel scared about my future.” Responses were rated from 1 (strongly disagree) to 4 (strongly agree), with 2 reverse-coded items; higher average scores indicated higher levels of hope.

#### Empowerment Scale

The Empowerment Scale is a 28-item measure developed with individuals experiencing serious mental illness to assess perceived self-agency, control, and confidence. It has strong psychometric properties, including high internal consistency (*α*=.81), and demonstrated construct validity [16]. Sample questions include “I can pretty much determine what will happen in my life” and “People are only limited by what they think is possible.” Items were scored from 1 (strongly agree) to 4 (strongly disagree), with lower scores reflecting higher levels of empowerment.

#### Flourishing Scale

The Flourishing Scale measures psychological well-being across domains such as relationships, self-esteem, purpose, and optimism. This 8-item scale has demonstrated excellent reliability (*α*=.87-.91) and strong associations with other mental health indicators [17]. Sample items include “I lead a purposeful and meaningful life” and “I am competent and capable in the activities that are important to me.” Response options ranged from 1 (strongly disagree) to 7 (strongly agree), with total scores from 8 to 56; higher scores indicated greater psychological resources.

#### Mindful Attention Awareness Scale

The Mindful Attention Awareness Scale is a 15-item scale that assesses dispositional mindfulness through present-moment attention and awareness. It has high internal consistency (*α*=.82-.87) and strong validity across diverse populations [18]. Example items include “I could be experiencing some emotion and not be conscious of it until sometime later” and “I tend not to notice feelings of physical tension or discomfort until they really grab my attention.” Items were rated from 1 (almost always) to 6 (almost never), with higher average scores reflecting greater mindfulness.

#### Active-Empathic Listening Scale

This scale evaluates the listener’s emotional and cognitive engagement during interpersonal communication. The 11-item Active-Empathic Listening Scale is psychometrically sound, with high internal consistency (*α*=.80-.90) [19]. Sample questions include “I am sensitive to what others are not saying” and “I show others that I am listening by my body language.” Items were scored on a 7-point scale from 1 (never or almost never true) to 7 (always or almost always true), with higher average scores indicating higher levels of active-empathic listening skills.

#### Social Connectedness Scale-Revised

The revised Social Connectedness Scale assesses an individual’s sense of belonging and connection to others. This 20-item scale has been widely used, with strong internal consistency (*α*=.91) [20], although some psychometric limitations, such as response bias, have been noted [21]. Sample questions include “I feel distant from people” and “I find myself actively involved in people’s lives.” Items were scored from 1 (strongly disagree) to 6 (strongly agree), with negatively worded items reverse-coded; higher total scores indicated greater social connectedness.

#### Positive and Negative Affect Schedule

Positive and Negative Affect Schedule (PANAS) is a 20-item scale used to measure emotional states, including both positive and negative affect. The scale has excellent reliability (positive affect *α*=.86-.90; negative affect *α*=.84-.87) and strong construct validity [22]. Participants were asked how often they felt emotions like “interested,” “distressed,” and “ashamed” in the past week, using a 5-point scale from 1 (very slightly or not at all) to 5 (very much). Positive and negative items were summed separately; higher scores indicated higher levels of the respective affect.

#### UCLA 3-Item Loneliness Scale

This brief 3-item scale measures perceived social isolation and loneliness and is derived from the longer 20-item University of California Los Angeles (UCLA) Loneliness Scale. It has acceptable reliability (*α*=.72) and strong correlations with longer loneliness assessments [23]. Sample items include “How often do you feel that you lack companionship?” and “How often do you feel left out?” Items were rated from 1 (hardly ever) to 3 (often), with lower average scores reflecting lower loneliness levels.

#### Emotional CPR Scale

The Emotional CPR scale consists of items assessing beliefs and attitudes related to emotional support, presence, nonverbal communication, and self-care when supporting others in distress.

### Reliability Analysis of Measures

The reliability of each scale was assessed using Cronbach *α*. Reliability analysis was conducted to determine the reliability and internal consistency of presurvey and postsurvey Emotional CPR, UCLA Loneliness Scale, Revised Social Connectedness Scale, Empowerment Scale, PANAS, Active-Empathic Listening Scale, Flourishing Scale, Mindful Attention Awareness Scale, and Herth Hope Scale. The results indicate that all presurvey and postsurvey scales have acceptable to excellent reliability and internal consistency since Cronbach *α* coefficients ranged from .63 to .91 (Table 1).

**Table 1.** Internal consistency and reliability (Cronbach *α*) of pretraining and posttraining measures (N=85).

Scale	Pretraining *α*	Posttraining *α*
Emotional CPR^a^	.68	.66
UCLA^b^ Loneliness Scale	.79	.77
Social Connectedness Scale-Revised	.71	.90
Empowerment Scale	.77	.73
Positive and Negative Affect Schedule	.66	.71
Active-Empathic Listening Scale	.63	.64
Flourishing Scale	.91	.87
Mindful Attention Awareness Scale	.77	.72
Herth Hope Scale	.88	.89

a Emotional CPR: Emotional Connection, Empowering, Revitalizing.

b UCLA: University of California Los Angeles.

### Quantitative Data Analysis

Descriptive statistics were calculated to summarize participant characteristics. Internal consistency and reliability of each scale was assessed using Cronbach *α*. Pretraining to posttraining changes across outcome variables were examined using paired-samples 2-tailed *t* tests, and effect sizes were calculated using Cohen *d*. Cohen *d* values reflect the direction and magnitude of change from pretraining to posttraining, with positive values indicating higher posttraining scores and negative values indicating lower posttraining scores; effect sizes were interpreted using conventional benchmarks (0.20 for small, 0.50 for medium, and 0.80 for large), with scale directionality considered in interpreting substantive meaning. To explore potential gender differences over time, repeated-measures ANOVA were conducted with time (pre vs post) as the within-subject factor and gender as the between-subject factor. Statistical significance was set at *P*<.05. All analyses were conducted using IBM SPSS Statistics.

### Qualitative Data Collection

Two focus group discussions were conducted through Zoom, with the first session held on January 6, 2025 (n=10, 59%) and the second on January 13, 2025 (n=7, 41%). Each session lasted approximately 60 minutes and was audio-recorded for accuracy. The lead researcher conducted both focus group discussions, and a professional transcription service was used to transcribe the recordings.

A semistructured interview guide was used to explore participants’ experiences with the Emotional CPR training, covering the following questions:

1. How has the Emotional CPR training influenced your sense of empowerment?
2. In what ways has the training affected your ability to practice empathetic listening?
3. What impact has the training had on your well-being and overall sense of flourishing?
4. Did the training influence your sense of hope or optimism for the future?
5. How has the training shaped your social interactions and relationships?
6. Did Emotional CPR training alter your perspective on emotional distress, either in yourself or in others?
7. In what ways did the training address emotional challenges linked to systemic issues?
8. How has Emotional CPR training influenced your self-perception?
9. How do you anticipate applying the knowledge and skills gained from Emotional CPR moving forward?
10. What suggestions do you have for improving the effectiveness of Emotional CPR training?

The interview guide included questions aligned with the training objectives, which may have shaped or directed participants’ responses toward specific domains of interest.

### Qualitative Data Analysis

A reflexive thematic analysis informed by phenomenological principles [24] was used to analyze qualitative data. While the analysis emphasized participants’ lived experiences and meaning-making, it also incorporated both inductive coding and deductive attention to domains reflected in the interview guide and training objectives. Transcripts were independently coded by 2 members of the research team using an iterative thematic process. Coding discrepancies were discussed and resolved through consensus to enhance credibility and dependability. Themes were generated through a reflexive and iterative analytic process that prioritized participants’ language and interpretations while situating findings within the context of the training content. The integration of quantitative and qualitative strands is summarized in Table 2.

**Table 2.** Integration of quantitative and qualitative data.

Component	Quantitative strand	Qualitative strand	Integration purpose
Focus	Measured changes in self-reported well-being and interpersonal skills	Explored participants’ lived experiences, perceptions, and meaning-making related to Emotional CPR^a^ training	Developed a comprehensive understanding of measurable outcomes and experiential impact
Analysis approach	Statistical analyses conducted independently	Reflexive thematic analysis informed by phenomenological inquiry; inductive coding and iterative theme development	Findings compared at the interpretation stage to examine convergence, complementarity, and divergence
Key contribution	Identified statistically significant pre-post changes associated with the training	Illuminated how participants interpreted and contextualized those changes within personal and community contexts	Enhanced interpretive depth and clarified mechanisms underlying observed quantitative outcomes
Design type	Convergent mixed methods	Convergent mixed methods	Integration strengthened validity through triangulation and enriched understanding of training impact

a Emotional CPR: Emotional Connection, Empowering, Revitalizing.

Building on this approach, the analysis was designed to systematically identify patterns across participants’ accounts while remaining grounded in the exploration of lived experience and meaning-making [24,25]. It focused on interpreting how participants described, understood, and made sense of their experiences with Emotional CPR training. A reflexive 6-phase thematic analysis process guided the analytic procedures [25]:

1. Data preparation and familiarization: Audio recordings were transcribed verbatim, and identifying details were removed to ensure confidentiality. Transcripts were imported into NVivo 12 to support data management. Members of the research team read and reread the transcripts to become deeply familiar with the content, noting preliminary impressions and experiential insights.
2. Generating initial codes: Transcripts were systematically coded to capture significant features of participants’ lived experiences. Coding was primarily inductive and data-driven, while also informed by the domains reflected in the interview guide and training objectives.
3. Constructing themes: Codes were examined for patterns and grouped into candidate themes that reflected shared dimensions of experience. Attention was given to both semantic content and underlying meanings.
4. Reviewing and refining themes: Themes were reviewed in relation to the coded data and the full dataset to ensure coherence, distinction, and fidelity to participants’ accounts. Redundant or unclear categories were refined or collapsed.
5. Defining and naming themes: Each theme was clearly defined to articulate its central organizing concept and its relevance to the broader experiential narrative.

### Trustworthiness of the Qualitative Findings

Several steps were taken to strengthen the rigor of the qualitative analysis. Two members of the research team independently reviewed and coded the transcripts, meeting regularly to discuss differences and refine themes until consensus was reached. Including direct participant quotations in the *Results* section allows readers to see how interpretations are grounded in participants’ own words.

The analytic process followed a structured and documented approach. Coding decisions and theme development were tracked throughout the analysis to ensure consistency and transparency. This process helped maintain alignment between the raw data and the final themes.

Reflexivity was also an important part of the analytic process. The lead researcher identifies as Black, which was particularly relevant given the focus of the study on BIPOC participants and facilitators. This shared cultural context contributed to building trust during focus groups and may have shaped how participants felt comfortable expressing their experiences. Throughout the analysis, care was taken to remain attentive to how lived experience and insider perspective could influence interpretation, ensuring that themes reflected participants’ narratives rather than researchers’ assumptions.

### Integration of Quantitative and Qualitative Data

Qualitative themes were then examined alongside quantitative results to provide a comprehensive interpretation of the training’s impact. This integrative step allowed experiential insights to contextualize and deepen the understanding of measurable outcomes.

By using thematic analysis informed by phenomenological inquiry, the study generated an interpretive account of participants’ lived experiences rather than seeking to identify universal or essentialized structures of consciousness, defined here as fixed or generalizable features of experience. The findings highlight how Emotional CPR training shaped participants’ perceptions of personal growth, relational connection, and emotional resilience within the BIPOC community.

## Results

### Descriptive Statistics

A total of 85 participants completed both pretraining and posttraining surveys. Of these, 65 (76%) identified as female participants, 17 (20%) as male participants, 2 (2%) as another gender, and 1 (1%) as Two-Spirit, transgender, gender-nonconforming, or nonbinary. Most participants were identified as Black or African American (70/85, 82%) and were employed full-time (64/85, 75%). The mean age of participants was 41.93 (SD 11.21; range 19‐69) years (Table 3).

**Table 3.** Sociodemographic characteristics of survey participants (N=85).^a^

Characteristic	Values, n (%)
Gender	
Male	17 (20)
Female	65 (76)
Other	2 (2)
Two-Spirit/transgender/gender-nonconforming/nonbinary	1 (1)
Race	
Black or African American	70 (82)
White	1 (1)
American Indian or Alaska Native	1 (1)
Asian	2 (2)
Black or African American and more than 1 race	1 (1)
Asian and more than 1 race	1 (1)
More than 1 race	9 (11)
Hispanic or Latino ethnicity	
Yes	11 (13)
No	74 (87)
Employment status	
Full-time	64 (75)
Full-time and other roles	1 (1)
Part-time	4 (5)
Volunteer	5 (6)
Unemployed	8 (9)
Retired	3 (4)

a Percentages are rounded to whole numbers because N<100.

Seventeen participants participated in the focus group discussions. Of these, 14 (82%) were identified as female participants and 3 (18%) were identified as male participants (Table 4).

**Table 4.** Sociodemographic characteristics of focus group participants (N=17).^a^

Characteristic	Values, n (%)
Male	3 (18)
Female	14 (82)

a Percentages are rounded to whole numbers because N<100.

Additional demographic characteristics beyond gender were not systematically collected for focus group participants, which limits the ability to further contextualize the qualitative findings.

### Emotional CPR Item-Level Changes

Paired-samples *t* tests examined pretraining to posttraining changes in Emotional CPR scale items (Table 5). Significant increases were observed in items reflecting solution-focused support (*P*<.001), uncertainty in helping someone in distress (*P*<.001), and attempts to solve others’ problems (*P*<.001). Significant decreases were observed in items related to being with another person (*P*<.001), understanding nonverbal communication (*P*<.001), emotional self-care (*P*<.001), and openness to new approaches (*P*=.008). No significant changes were observed in emotional awareness or trauma recognition.

**Table 5.** Paired-samples *t* tests for Emotional CPR (Emotional Connection, Empowering, Revitalizing) item-level changes (N=85).

Item	Pretraining, mean (SD)	Posttraining, mean (SD)	*P* value	Cohen *d*
Emotional distress awareness	2.62 (0.93)	2.50 (1.05)	.21	0.14
Support—solving problems	2.95 (1.17)	3.40 (1.22)	<.001	–0.41
Support—being with someone	2.75 (1.02)	1.99 (1.09)	<.001	0.80
Trauma awareness	1.36 (0.53)	1.31 (0.51)	.20	0.14
Want to help but unsure how	2.81 (0.92)	3.28 (1.10)	<.001	–0.44
Emotional identification	1.85 (0.76)	1.76 (0.78)	.40	0.09
Sitting with strong emotions	1.48 (0.65)	1.44 (0.63)	.44	0.08
Sharing emotions	1.99 (0.97)	1.96 (0.94)	.77	0.03
Problem-solving tendency	2.60 (1.01)	3.32 (1.11)	<.001	–0.69
Meaning of “being with”	2.23 (0.89)	1.62 (0.75)	<.001	0.75
Self-nonverbal awareness	1.86 (0.68)	1.48 (0.59)	<.001	0.55
Comfort listening	1.63 (0.71)	1.39 (0.60)	<.001	0.43
Self-care before or after distress	2.26 (0.90)	1.86 (0.86)	<.001	0.45
Openness to new ideas	1.53 (0.68)	1.35 (0.63)	.008	0.29
Recognizing others’ nonverbal cues	1.86 (0.71)	1.54 (0.66)	<.001	0.44

### Scale-Level Outcomes

Significant pretraining to posttraining changes were observed across several standardized measures (Table 6). Loneliness scores decreased (*P*=.02), and positive affect increased (*P*=.002). Significant declines were observed in empowerment (*P*=.03), active-empathic listening (*P*=.02), flourishing (*P*=.02), and hope (*P*<.001). No statistically significant changes were observed in social connectedness or mindfulness.

**Table 6.** Pretraining to posttraining changes across outcome measures (N=85).

Measure	Pretraining, mean (SD)	Posttraining, mean (SD)	*P* value	Cohen *d*
UCLA^a^ Loneliness Scale	5.48 (1.71)	5.22 (1.46)	.02	0.25
Social Connectedness Scale-Revised	65.85 (6.62)	65.78 (5.92)	.90	0.01
Empowerment Scale	51.90 (6.51)	50.68 (6.03)	.02	0.26
PANAS^b^ (positive affect)	55.14 (8.27)	58.15 (8.32)	.002	–0.37
Active-Empathic Listening Scale	12.88 (4.30)	11.89 (4.19)	.02	0.26
Flourishing Scale	16.04 (6.72)	14.77 (6.09)	.02	0.27
Mindful Attention Awareness Scale	7.64 (2.43)	7.72 (2.27)	.66	–0.05
Herth Hope Scale	19.57 (3.23)	18.51 (3.04)	<.001	0.41

a UCLA, University of California Los Angeles.

b PANAS: Positive and Negative Affect Schedule.

### Qualitative Study Results

Qualitative analysis of focus group discussions identified 6 primary themes and related subthemes. Themes and representative participant quotations are presented in Table 7.

**Table 7.** Themes and representative quotations from focus group discussions (N=17).

Themes and subtheme	Representative quotations
Empowerment through training	
Increased confidence	“I felt very empowered afterwards.” “I definitely felt more confident in my approaches and how I want to engage with others.”
Awareness of biases	“It diversified the lens and scope of which I understand and listen to the stories of others.”
Personal growth	“It was a great growth opportunity for me.” “It helped me ground myself to being a lot more positive.”
Active listening and empathy	
Improved listening	“People need your ears, not always your mouth.” “It taught me not to listen to respond but to truly listen to understand.”
Nonverbal communication	“I’m mindful of my body language.” “My skills in nonverbal connection with others have improved.”
Managing personal triggers	“I have to check my personal experience because it could affect my ability to support others.”
Emotional impact of training	
Emotional vulnerability	“Just sitting in silence and holding space can sometimes be the most effective support.” “Being vulnerable without walls was important.”
Self-care	“Put that oxygen mask on first.”
Shared emotional experiences	“It let me know I wasn’t alone in how I was feeling.”
Building social connections	
Cultural safety and trust	“BIPOC^a^ facilitators created a safe space.” “Trust is a big thing in BIPOC communities.”
Community connection	“The relationships built within the group had a strong sense of community.”
Application of skills in daily life	
Personal application	“The training helped me parent better and connect with my child’s emotions.”
Professional application	“I use this in my personal life and at work with clients experiencing mental health challenges.”
Training structure and recommendations	
Need for more time	“More time would allow more role-playing and sharing.”
Ongoing support	“A follow-up group would help reinforce skills and allow people to practice more.”

a BIPOC: Black, Indigenous, and people of color.

### Qualitative Findings

Qualitative analysis of focus group discussions identified 6 primary themes: (1) empowerment through training, (2) active listening and empathy, (3) emotional impact of training, (4) building social connections, (5) application of skills in daily life, and (6) training structure and recommendations. Representative quotations are presented in Table 7.

Empowerment through training included increased confidence, greater awareness of personal biases, and personal growth. Participants described feeling more prepared to engage with others in emotionally supportive ways and more aware of how their communication patterns and assumptions influenced interactions.

Active listening and empathy reflected shifts in how participants approached conversations. Many emphasized learning to listen with intention rather than focusing on responding or solving problems. Participants also described an increased awareness of nonverbal communication and acknowledged the need to manage personal emotional triggers when supporting others.

The theme Emotional impact of training captured experiences of vulnerability, self-reflection, and self-care. Participants described emotionally meaningful discussions, particularly within culturally affirming spaces. The importance of maintaining personal boundaries and engaging in self-care when emotionally activated was also emphasized.

Building social connections highlighted the role of cultural safety and trust in shaping the training experience. Participants noted that having BIPOC facilitators contributed to a sense of openness and shared understanding. Several participants described developing a stronger sense of community during the sessions.

Under Application of skills in daily life, participants reported using training principles in family interactions, professional settings, and peer relationships. Skills such as asking whether someone wanted listening or advice, maintaining boundaries, and being present in conversations were described as particularly useful.

Finally, Training structure and recommendations included suggestions for additional practice time, in-person options, follow-up sessions, and broader dissemination of training to professionals and youth populations.

### Integration of Qualitative Themes With Quantitative Findings

The integration of qualitative and quantitative findings provides a more comprehensive understanding of how participants experienced the training. While quantitative analyses demonstrated reductions in loneliness and increases in positive affect, they also showed modest declines in empowerment, hope, flourishing, and active-empathic listening.

These qualitative themes provide context for the quantitative findings, particularly the observed declines in empowerment, hope, and active-empathic listening. Participants described increased self-awareness and emotional vulnerability following training, which may have temporarily influenced their self-evaluations of competence and confidence.

Reductions in loneliness were consistent with qualitative themes of Building Social Connections and Emotional Impact of Training. Participants described increased feelings of connection, cultural safety, and shared understanding within the training space. These relational experiences align with the observed decrease in self-reported loneliness.

In contrast, empowerment scores declined slightly, despite participants describing experiences of increased confidence and personal growth. Qualitative data suggest that the training may have heightened awareness of the complexities involved in supporting others, including recognition of personal biases and emotional triggers. This increased self-awareness may have influenced participants’ posttraining self-assessments, resulting in more cautious ratings.

A similar pattern was observed for active-empathic listening. While participants described a deeper understanding of “being with” someone rather than solving problems, they also acknowledged the emotional effort required to practice these skills. This suggests that increased awareness of the challenges involved in sustained empathetic engagement may have contributed to modest declines in self-reported listening scores.

Declines in hope and flourishing may also be understood in light of the emotionally vulnerable discussions described in the qualitative findings. Participants engaged in dialogue related to trauma, systemic racism, and lived experiences. Although these conversations fostered connection and validation, they may have also surfaced unresolved stressors, temporarily influencing perceptions of well-being.

Taken together, the integrated findings suggest that the training promoted relational connection and emotional awareness while simultaneously increasing reflection on personal limitations and systemic realities. These processes may explain the combination of relational gains and short-term shifts in self-perceived empowerment and well-being observed in the quantitative data.

## Discussion

### Principal Findings

This study examined pretraining to posttraining changes among BIPOC participants who completed Emotional CPR facilitated by BIPOC trainers. Using a convergent mixed methods design, we integrated quantitative and qualitative findings to better understand participants’ emotional and interpersonal experiences following the training.

Quantitatively, participants reported reduced loneliness and increased positive affect immediately after training. These findings were supported by qualitative accounts describing feelings of connection, validation, and cultural safety. Participants frequently emphasized that being in a space led by BIPOC facilitators fostered trust and openness. Statements such as “It let me know I wasn’t alone in how I was feeling” reflect the relational environment that likely contributed to decreased loneliness. These results are particularly meaningful given the systemic challenges and racial stressors that affect BIPOC communities, where culturally resonant spaces for connection can promote belonging and support [26].

At the same time, modest declines were observed in empowerment, active-empathic listening, flourishing, and hope. Although participants described increased confidence and emotional awareness, qualitative findings suggest that the training increased recognition of emotional complexity, personal triggers, and systemic barriers. Greater self-awareness may have influenced posttraining self-ratings, resulting in more cautious evaluations of competence. Conversations about racism and systemic inequities can evoke strong emotional responses and may temporarily affect perceptions of agency [4].

Similarly, declines in active-empathic listening may reflect heightened awareness of the effort required to sustain emotional presence. Participants acknowledged that deep listening requires emotional regulation and self-reflection. This aligns with the literature on emotional labor, which suggests that engaging in emotionally intensive dialogue can affect one’s capacity for sustained empathic engagement [27]. While positive affect increased, mindfulness and overall social connectedness did not change significantly, suggesting that while the training fostered immediate relational gains, the longer-term integration of skills into daily life may require continued reinforcement.

Declines in hope and flourishing also warrant attention. Participants described engaging in emotionally vulnerable conversations about trauma and lived experiences. While these dialogues fostered connection and validation, they may have simultaneously surfaced unresolved stressors. Research suggests that confronting systemic realities can influence hope and perceptions of well-being, particularly in marginalized communities [2,28]. Recent work on radical healing frameworks emphasizes that processes of naming and processing racial trauma may initially surface emotional strain before fostering longer-term resilience [28]. These findings highlight the complexity of short-term emotional shifts following reflective, trauma-informed interventions.

### Comparison With Prior Work

These findings extend previous research on peer-led mental health interventions. Prior studies of peer support models demonstrate improvements in empowerment, engagement, and emotional well-being [5-8]. Recent knowledge syntheses further highlight that peer-based models strengthen relational continuity and trust within community mental health systems [8]. Similarly, earlier studies of Emotional CPR and peer-delivered interventions have demonstrated feasibility and preliminary effectiveness in community-based settings [10,11,29]. This study builds on this work by examining a culturally responsive implementation facilitated entirely by BIPOC trainers for BIPOC participants.

The reduction in loneliness aligns with the literature emphasizing the importance of culturally responsive spaces in fostering belonging and relational safety [26]. Increases in positive affect are also consistent with research linking emotional connection to resilience and stress buffering [30,31]. At the same time, the observed declines in empowerment and hope highlight that reflective and trauma-informed interventions may produce complex, nonlinear outcomes. Literature on emotional labor and counselor listening exhaustion suggests that engaging deeply with distress can temporarily affect self-perceived competence [27].

The findings also underscore calls for culturally tailored mindfulness and emotional regulation strategies within BIPOC communities [28]. While mindfulness scores did not change significantly, participants described increased awareness and reflection, suggesting potential areas for future adaptation.

### Limitations

Several limitations should be considered. First, the study used a single-group pre-post design without a comparison group, limiting causal inference. Observed changes cannot be attributed solely to the intervention. Second, outcomes were measured immediately posttraining, and longer-term effects remain unknown. Third, all measures were self-reported and may be influenced by social desirability or momentary emotional states.

The sample consisted primarily of Black or African American participants and women, which may limit generalizability to other populations or BIPOC subgroups. Additional demographic characteristics of focus group participants were not collected, which limits the ability to contextualize qualitative findings across participant backgrounds. Additionally, participants had access to internet-enabled devices and were able to participate in web-based training, which may exclude individuals with fewer resources. Finally, while qualitative integration strengthened interpretation, future studies using longitudinal designs and control groups would provide more robust evidence.

### Conclusions

Participation in a culturally responsive, peer-led Emotional CPR training facilitated by BIPOC trainers was associated with short-term reductions in loneliness and increases in positive affect, alongside modest declines in self-rated empowerment, hope, flourishing, and active-empathic listening. Qualitative findings suggest that increased emotional awareness and engagement with systemic stressors may influence short-term self-evaluations of competence and well-being.

These results highlight the importance of culturally responsive peer-led spaces in fostering connection and emotional safety, while also underscoring the need for follow-up support, mentorship, and continued skill practice to sustain empowerment and resilience over time. Future research should examine longer-term outcomes and explore strategies for reinforcing gains in culturally responsive mental health interventions.

## Abbreviations

BIPOC: Black, Indigenous, and people of color
Emotional CPR: Emotional Connection, Empowering, Revitalizing
PANAS: Positive and Negative Affect Schedule
